# Peripartum outcomes and immune responses after SARS-CoV-2 infection in the third trimester of pregnancy

**DOI:** 10.1186/s12884-024-06707-5

**Published:** 2024-07-24

**Authors:** Qi Shen, Shuai Dong, Neelam Kumari Shah, Yuan Liang, Jie Wang, Yan-Hong Shan, Jin He

**Affiliations:** Department of obstetrics, Obstetrics and Gynaecology Center, The First Hospital of Jilin University, Changchun, 130061 China

**Keywords:** COVID-19, Pregnancy, Peripartum outcomes, Immune response

## Abstract

**Background:**

SARS-CoV-2 infection in pregnant women during the third trimester resulted in overall adverse pregnancy outcomes compared to non-infected controls and a unique humoral and cellular response at delivery. In this study we aimed to assess the impact of SARS-CoV-2 infection on maternal/neonatal peripartum outcomes andimmunological profiles.

**Method:**

In this study, we recruited 304 SARS-CoV-2 infected pregnant women and 910 SARS-CoV-2 non-infected pregnant women who were admitted for delivery. Peripartum and neonates’ outcomes response to SARS-CoV-2 infection were analyzed. Furthermore, we characterized the antibody and cytokines profile in SARS-CoV-2 infected maternal blood (MB) and cord blood (CB). We also assessed routine laboratory tests and liver function tests in MB before labor. Unpaired T test, Mann-Whitney test and Spearman test were used to analyze the data.

**Results:**

SARS-CoV-2 infected pregnant women were significantly associated with increased risk of adverse pregnancy outcomes, including preterm labor (13.8% vs. 9.5%, *p* = 0.033) and meconium-stained amniotic fluid (8.9% vs. 5.5%, *p* = 0.039). The risk of low birth weight (< 2500 g) (10.5% vs. 6.5%, *p* = 0.021) and Apgar score < 8 at 1-minute (9.2% vs. 5.8%, *p* = 0.049) significantly increased in newborns from COVID-19 positive mothers than their counterparts. Our results showed that antibodies were increased in adverse-outcome SARS-CoV-2 infected mothers and their neonates, and abnormal proportion of immune cells were detected in SARS-CoV-2 infected mothers. While the immune response showed no difference between adverse-outcome infected pregnant women and normal-outcome infected pregnant women. Thus, SARS-CoV-2 infection during the third trimester of pregnancy induced a unique humoral and cellular response at delivery.

**Conclusion:**

SARS-CoV-2 infection closer to delivery could incline to adverse pregnancy outcomes. Therefore, the utmost care is required for SARS-CoV-2 infected pregnant women and their newborns.

**Trial registration:**

The study protocol was approved by the Institutional Review Board of the First Hospital of Jilin University with the approval code number 23K170-001, and informed consent was obtained from all enrolled patients prior to sample collection.

**Supplementary Information:**

The online version contains supplementary material available at 10.1186/s12884-024-06707-5.

## Background

Pregnant women are considered more susceptible to COVID-19 due to alterations in their immune system and cardiorespiratory physiology, which might lead to an altered immune response to SARS-CoV-2 infection in pregnancy. As pregnant women and their neonates have a unique interdependent immune system, the effect of COVID-19 on pregnant women during and after pregnancy and on their neonates is of great concern. Pregnant women with COVID-19 are at higher risk for hospitalization, ICU admission, sepsis, and even death as compared to their non-pregnant counterparts [1]. Previous studies have shown that pregnant women with COVID-19 are more likely to undergo obstetrical complications, such as pre-eclampsia, gestational diabetes, cesarean delivery, preterm birth, intrauterine growth restriction, and stillbirth [2–5]. During pregnancy, SARS-CoV-2 infection might present with different degrees of clinical manifestations, ranging from asymptomatic or only experiencing mild symptoms to developing severe/critical disease [6, 7]. Severe COVID-19 patients exhibited disordered immune responses, which triggers a cytokine storm that eventually leads to an increment of pro-inflammatory cytokines. These cytokine storms were found to mediate inflammatory reactions in the lung and failed to eliminate the pathogens [8, 9]. Currently, it is reported that pro-inflammatory cytokines in SARS-CoV-2 infected pregnant women can cross the placental barrier and have deleterious effects on the offspring [10]. However, due to the difference in sample number, the timing of infection, and the severity of symptoms, the obstetric and neonatal consequences, cytokine expression level and its increase trend are inconsistent in different literatures.

Herein, we investigated maternal and neonatal outcomes related to COVID-19 infected during the third trimester. Then, we employ a multidisciplinary strategy that incorporates the detection of SARS-CoV-2 specific antibodies (IgM and IgG), multiplex cytokine assays and laboratory tests to evaluate the association between maternal/neonatal immune response and the outcome of SARS-CoV-2 infection.

## Method

### Study design, participant recruitments and sample collection

**Fig. 1:**
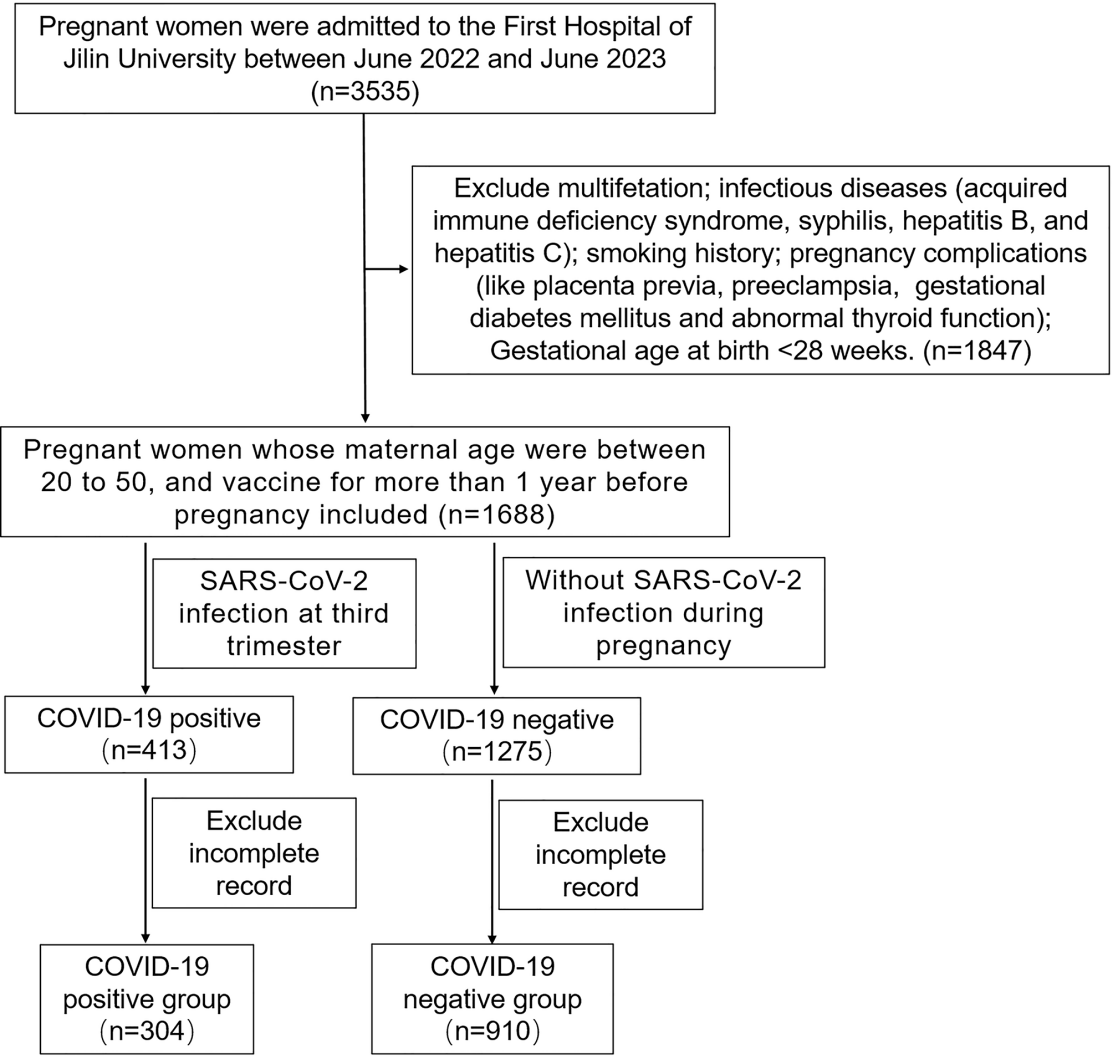
Flow diagrams of study participants inclusion

This cross-sectional study was conducted by the Critical Maternal Treatment Center of the First Hospital of Jilin University between June 2022 and June 2023 to explore the impact of SARS-CoV-2 infection on obstetric and neonatal outcomes. The sample population consisted of 304 SARS-CoV-2 infected pregnant patients who were admitted for delivery. The diagnostic criteria for SARS-CoV-2 infection during the third trimester were: (i) positive SARS-CoV-2 real-time RT-PCR during the third trimester [11]; (ii) at least three negative SARS-CoV-2 real-time RT-PCR, neither fever nor any respiratory symptoms with no history of close contact with SARS-CoV-2 infected people during the first and second trimester. 910 pregnant women who did not infect by SARS-CoV-2 and planned to deliver were offered enrollment into the control group. Pregnant women with a singleton gestation, age ranging from 20 to 50 years, no smoking history, no infection (e.g. acquired immune deficiency syndrome, syphilis, hepatitis B, and hepatitis C), and absence of pregnancy complications like placenta previa, preeclampsia, gestational diabetes mellitus and abnormal thyroid function during pregnancy were recruited in this study (Fig. 1). A survey was developed for this study (Supplementary file). The study protocol was approved by the Institutional Review Board of the First Hospital of Jilin University with the approval code number 23K170-001, and informed consent was obtained from all enrolled patients prior to sample collection. Studies were performed in accordance with the Declaration of Helsinki.

Maternal demographic characteristics (Maternal age, place of residence, mode of delivery, gravidity, parity, medical comorbidities and COVID-19 vaccination status), routine blood tests (WBC, RBC, HGB, Neutrophil, Lymphocyte etc.), other laboratory results (ALT, AST, SARS-CoV-2 RNA testing results), pregnancy complications (preterm delivery, fetal growth restriction etc.) as well as obstetric and neonatal outcomes, were abstracted from the electronic medical records or recorded in a questionnaire.

Maternal peripheral blood was collected at delivery, and umbilical cord blood was collected immediately after delivery into tubes with an anticoagulant (EDTA or citrate) and tubes without an anticoagulant. Blood samples were processed on the same day of collection from patients. Blood samples were collected into evacuated blood collection tubes without an anticoagulant and centrifuged at 1000 x g for 10 min at 4 °C. After centrifugation, the serum was aliquoted into cryogenic vials (Corning Incorporated, USA) and stored at − 80 °C for subsequent analysis of cytokines and antibody levels. To avoid the impact of an uncertain COVID-19 infection on serum testing, the negative control of MB serum and CB serum collected before 2019 were obtained from the Department of Biobank, Division of Clinical Research, The First Hospital of Jilin University.

### Determination of SARS-CoV-2 IgG and IgM antibodies

The IgG and IgM antibodies to RBD and Spike S1 antigens were measured in the serum samples of MB and CB using LEGEND MAX™ SARS-CoV-2 ELISA Kit (Biolegend, USA) according to the manufacturer’s instructions. Plates were read via Tecan Spark Microplate Reader (Tecan, Switzerland).

### Cytokine detection

Concentration of IL-6, MCP-1 (CCL2), G-CSF, IFN-α2, IL-2, IFN-γ, IL-7, IL-1RA, IL-8 (CXCL8), TNF-α, IP-10 (CXCL10), MIP-1α (CCL3), and IL-10 was assessed in the maternal and cord blood serum, according to the manufacturer’s instructions of LEGENDplex™ COVID-19 Cytokine Storm Panel 1 (Biolegend, USA). The data were read on a flow cytometry (BD Canto, USA). Finally, the data were analyzed using LEGENDplexTM Data Analysis Software.

### Statistical analysis

All statistical analyses were performed using GraphPad Prism, version 8.0 (GraphPad Software), and IBM SPSS Statistics software, version 27.0. Comparisons of antibody between the study groups were performed by the unpaired T test, and cytokine levels were performed by the Mann-Whitney test. Correlations of IgG antibody concentration in paired infected mothers and cord blood samples with adverse-outcome or normal-outcome were assessed by the Spearman test. We used logistic regression to estimate ORs for clinical data. *P* < 0.05 was considered statistically significant.

## Results

### SARS-CoV-2 infection lead to adverse peripartum and neonates’ outcomes

Our study recruited 1,214 participants, 304 SARS-CoV-2 infected pregnant women and 910 SARS-CoV-2 non-infected pregnant women from the First Hospital of Jilin University. Among the infected pregnant women, 304 patients were asymptomatic or presented with mild symptoms (e.g., fever and tachycardia), and only 2 patients were diagnosed as having severe symptoms of SARS-CoV-2 infection (requiring mechanical ventilation).

As displayed in Table 1, no statistically pronounced intergroup differences were observed for maternal age, place of residence, mode of delivery, gravidity, parity, medical comorbidities, and COVID-19 vaccination status (vaccination for more than one year before the onset of labor) (*P* > 0.1).

**Table 1:** Clinical characteristics of the study population

Clinical characteristic	COVID-19 positive *n* = 304	COVID-19 negative *n* = 910	X^2^	*P* ^a^
Maternal age, n (%)				
20–34 y old	216(71.1%)	688(75.6%)	2.483	0.115
35–50 y old	88(28.9%)	222(24.4%)		
Maternal age, (years)	32.49 ± 4.17	32.21 ± 3.95	-1.080^b^	0.280^b^
Place of residence, n (%)				
Urban	173(56.9%)	554(60.9%)	1.496	0.221
Rural	131(43.1%)	356(39.1%)		
Mode of delivery, n(%)				
Vaginal delivery	57 (18.8%)	160 (17.6%)	0.212	0.646
Cesarean delivery	247 (81.3%)	750 (82.4%)		
Gravidity, n (%)				
1	146(48.0%)	470(51.6%)	1.196	0.274
> 1	158(52.0%)	440(48.4%)		
Parity, n (%)				
1	217(71.4%)	678(74.5%)	1.148	0.284
> 1	87(28.6%)	232(25.5%)		
Medical comorbidities^↑^, n (%)				
Yes	12(3.9%)	26(2.9%)	0.893	0.345
No	292(96.1%)	884(97.1%)		
COVID-19 vaccination status, n (%)				
Received for more than 1 year	256(84.2%)	733(80.5%)	2.023	0.155
Not received	48(15.8%)	177(19.5%)		

^a^Comparisons between the groups were evaluated using the chi-square test.

^↑^Medical comorbidities: diabetes, hypertension, cardiovascular, kidney, or lung disease.

^b^Maternal age in cases and controls were compared as a continuous variable with T test.

We analyzed the common adverse pregnancy outcomes in the third trimester and the overall adverse pregnancy outcomes are the cumulative result listed in Table 2. After adjusting for all unrelated variables (Age and COVID-19 vaccination status were adjusted), infected pregnant women with SARS-CoV-2 in the third trimester showed an increased risk of overall adverse pregnancy outcomes (41.8% vs. 34.4%; adjusted OR = 1.355;95% CI: 1.038–1.769, *P* = 0.025). Specifically, women with COVID-19 were more likely to experience preterm labor (13.8% vs. 9.5%; adjusted OR = 1.535; 95% CI: 1.034–2.280, *P* = 0.033), and meconium-stained amniotic fluid (8.9% vs. 5.5%; adjusted OR = 1.671; 95% CI: 1.025–2.723, *P* = 0.039).

**Table 2:** Risk of adverse peripartum outcomes among COVID-19 positive patients and COVID-19 negative patients

Outcome	COVID-19 positive *n* = 304	COVID-19 negative *n* = 910	OR(95%CI), *P*	OR*(95%CI), *P*
Overall adverse pregnancy outcomes, n (%)	127(41.8%)	313(34.4%)	1.368(1.049–1.785), 0.021	1.355(1.038–1.769), 0.025
Preterm labor, n (%)	42(13.8%)	86(9.5%)	1.536(1.035–2.278), 0.033	1.535(1.034–2.280), 0.033
Meconium-stained amniotic fluid, n (%)	27(8.9%)	50(5.5%)	1.677(1.030–2.729), 0.038	1.671(1.025–2.723), 0.039
Prelabor rupture of membranes, n (%)	52(17.1%)	114(12.5%)	1.441(1.008–2.060), 0.045	1.427(0.997–2.041), 0.052
Fetal growth restriction, n (%)	8(2.6%)	10(1.1%)	2.432(0.951–6.220), 0.064	2.383(0.929–6.110), 0.071
Postpartum hemorrhage, n (%)	23(7.6%)	46(5.1%)	1.537(0.916–2.582), 0.104	1.582(0.940–2.662), 0.084
Adherent placenta, n (%)	26(8.6%)	67(7.4%)	1.177(0.733–1.888), 0.500	1.157(0.719–1.862), 0.542
Oligohydramnios, n (%)	7(2.3%)	19(2.1%)	1.105(0.460–2.655), 0.823	1.104(0.458–2.661), 0.826

* Age and COVID-19 vaccination status were adjusted. ICU: Intensive care unit. Overall adverse pregnancy outcomes contains one or more adverse pregnancy outcome listed in Table 2.

**Fig. 2:**
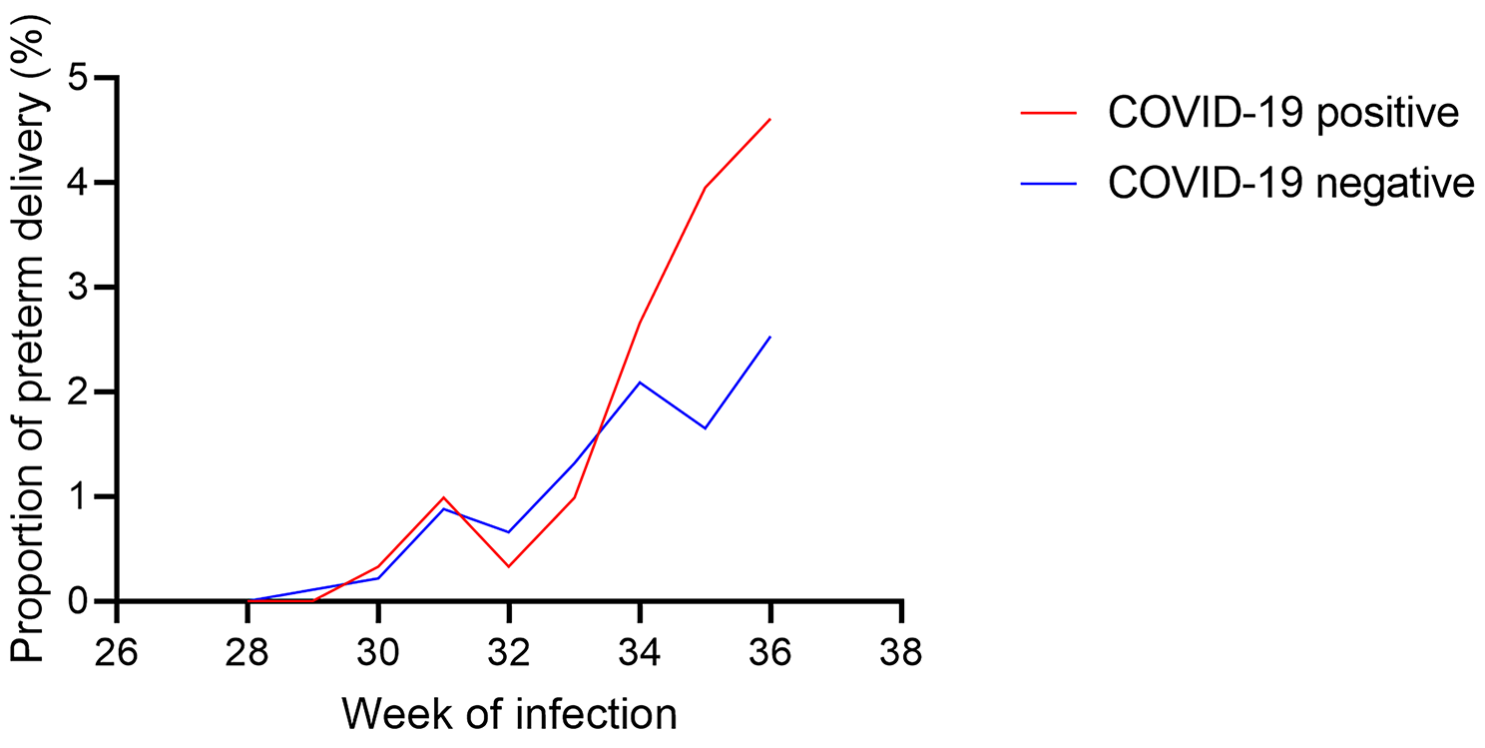
The proportion of preterm delivery after COVID‑19 according to week of infection. Red line represented COVID‑19 positive pregnant women, blue line represented COVID‑19 negative pregnant women

Furthermore, longer pregnancies are more likely to have been exposed to SARS-CoV-2, so we analyzed the association of different infection week with risks of preterm birth. As shown in Fig. 2, pregnant women infected by COVID-19 were more likely to trigger preterm birth at 34 week to 36 week.

Considering neonatal outcomes (Table 3), the incidence of low birth weight (< 2500 g) (10.5% vs. 6.5%; adjusted OR = 1.272;95% CI: 1.082–2.677, *P* = 0.021) and Apgar score < 8 at 1-minute (9.2% vs. 5.8%; adjusted OR = 1.580; 95% CI: 1.001–2.610, *P* = 0.049) were markedly increased in newborns from COVID-19 positive mothers than in newborns from COVID-19 negative mothers. Neonatal fever in COVID-19 positive mothers was similar with that in COVID-19 negative mothers. At 28 day after birth, the rate of low birth weight in neonates born by COVID-19 positive pregnant women with adverse outcome showed significantly difference compared to those with normal outcome pregnant women. There is no statistically significant difference between the two groups in terms of fever and newborn mental status (Table 4).

**Table 3:** Risk of neonatal outcomes born by COVID-19 positive mothers and COVID-19 negative mothers

Outcome	COVID-19 positive mother *n* = 304	COVID-19 negative mother *n* = 910	OR(95%CI), *P*	OR*(95%CI), *P*
Low birth weight (< 2500 g), n (%)	32(10.5%)	59(6.5%)	1.697(1.080–2.665), 0.022	1.272(1.082–2.677), 0.021
Apgar score < 8 at 1-minute, n (%)	28(9.2%)	53(5.8%)	1.640(1.018–2.645), 0.042	1.580(1.001–2.610), 0.049
Apgar score < 8 at 5-minute, n (%)	5(1.6%)	9(1.0%)	1.674(0.557–5.034), 0.359	1.648(0.546–4.971), 0.375
Neonatal fever, n (%)	22(7.2%)	49(5.4%)	1.261(0.754–2.111), 0.377	1.249(0.746–2.094), 0.398

* Age and COVID-19 vaccination status were adjusted.

**Table 4:** Risk of neonatal outcomes (28 days after birth) born by COVID-19 positive pregnant women with adverse outcome or normal outcome before labor

Characteristic	Adverse outcomes *n* = 127	Normal outcomes *n* = 177	OR(95%CI), *P*	OR*(95%CI), *P*
Low weight, (< 3000 g), n (%)	15(11.8%)	9(5.1%)	2.500(1.058–5.910), 0.037	2.477(1.009–6.082), 0.048
Fever, n (%)	10(7.9%)	12(6.8%)	1.175(0.491–2.811), 0.717	1.064(0.429–2.164), 0.893
Poor state, n (%)	8(6.3%)	5(2.8%)	2.313(0.738–7.242), 0.150	2.474(0.758–8.070), 0.133

*Age, place of residence, mode of delivery, gravidity, parity, medical comorbidities, and COVID-19 vaccination status were adjusted.

Poor state mainly included the newborn’s feeding status, activity levels, and urination and defecation status.

### Antibodies are increased in adverse-outcome SARS-CoV-2 infected mothers and their neonates

**Fig. 3:**
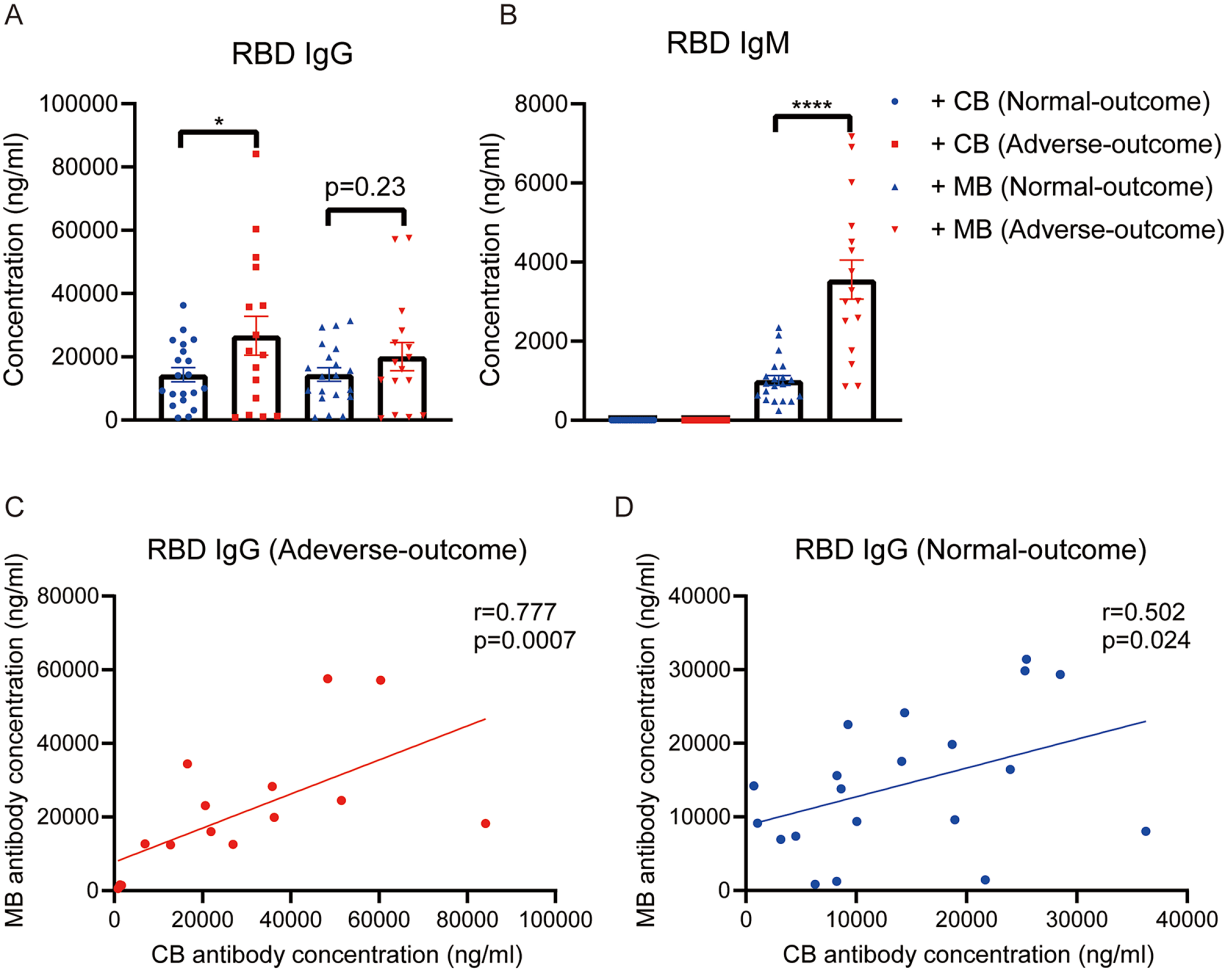
Anti-SARS-CoV-2 antibody levels in infected CB and MB. Comparison of RBD IgG **(A)** and RBD IgM **(B)**, serum levels in four groups: infected CB with normal-outcome (*n* = 20), infected CB with adverse-outcome (*n* = 16), infected MB with normal-outcome (*n* = 20) and infected MB with adverse-outcome (*n* = 16). Groups were compared by the unpaired T test. *p < 0.05. Correlations of IgG antibody concentration in paired infected mothers and cord blood samples with adverse-outcome **(C)** or normal-outcome **(D)** represented as a linear model and assessed by the Spearman test, showing the r and *p*-values

Spike (S) is the most crucial surface membrane protein of the SARS-CoV-2 virus, comprising two subunits, S1 and S2. S1 mainly contains a receptor-binding domain (RBD). We utilized RBD proteins to detect the levels of anti-SARS-CoV-2 specific antibodies. Both infected maternal blood (MB) serum and infected cord blood (CB) serum contained of RBD IgG (Fig. 3A). IgM detected in MB were correlated with the early stage of infection. Since the IgM antibody cannot penetrate the placental barrier, the RBD IgM antibody levels were undetected in CB serum (Fig. 3B).

Furthermore, among the infected pregnant women, 127 patients had adverse peripartum outcome, another 177 patients had normal peripartum outcome (Table 2). So we separated the infected pregnant women into two groups: normal-outcome samples (*n* = 20) and adverse-outcome samples (*n* = 16). Notably, the higher trend of IgM antibody levels in adverse-outcome MB was statistically pronounced than in normal-outcome MB, whereas the same trend of IgG antibody levels in MB was insignificant (Fig. 3A, B). The IgG antibody levels were also increased in adverse-outcome CB serum compared with normal-outcome CB serum. Moreover, either in adverse-outcome samples or in normal-outcome samples, infected MB serum showed positive significant correlations with paired infected CB serum on RBD IgG antibody concentrations (Fig. 3C and D).

### Cytokine profile is similar in adverse-outcome group and normal-outcome group

**Fig. 4:**
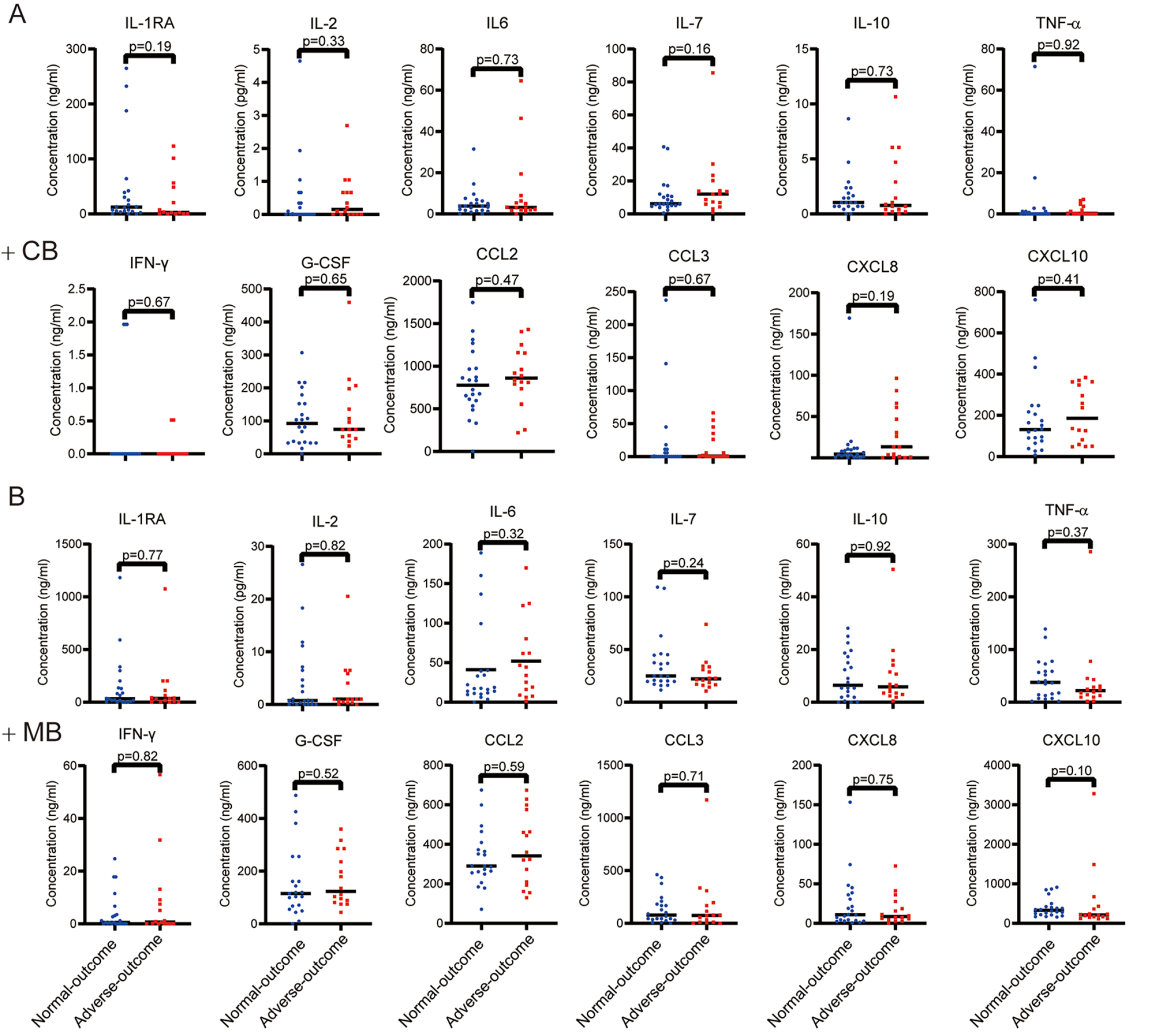
Cytokine and chemokine concentrations in SARS-CoV-2 infected MB and CB. Infected CB **(A)** and infected MB **(B)** were separated into normal-outcome (*n* = 20) and adverse-outcome (*n* = 16). Groups were compared by the Mann-Whitney test. Wilcoxon test was used to analyze the differences in the ratios of 12 cytokines between infected CB and infected MB: IL-1RA *p* = 0.07 (normal-outcome) *p* = 0.31 (adverse-outcome); IL-2: *p* = 0.02 (normal-outcome) *p* < 0.01 (adverse-outcome); IL-6: *p* < 0.01 (normal-outcome) *p* < 0.01 (adverse-outcome); IL-7: *p* < 0.01 (normal-outcome) *p* = 0.01 (adverse-outcome); IL-10: *p* < 0.01 (normal-outcome) *p* < 0.01 (adverse-outcome); TFN-α: *p* < 0.01 (normal-outcome) *p* < 0.01 (adverse-outcome); IFN-γ: *p* < 0.01 (normal-outcome) *p* < 0.01 (adverse-outcome); G-CSF: *p* = 0.32 (normal-outcome) *p* = 0.12 (adverse-outcome); CCL2: *p* < 0.01 (normal-outcome) *p* < 0.01 (adverse-outcome); CCL3: *p* < 0.01 (normal-outcome) *p* < 0.01 (adverse-outcome); CXCL8: *p* = 0.1 (normal-outcome) *p* = 0.32 (adverse-outcome); CXCL10: *p* < 0.01 (normal-outcome) *p* = 0.16 (adverse-outcome)

SARS-CoV-2 infection can lead to multi-organ damage with a cytokine storm in the systemic circulation [12]. Thus, we detected the systemic cytokine response in MB and CB serum by measuring the concentrations of 12 cytokines. Overall, the cytokine profile in the serum of adverse-outcome MB and adverse-outcome CB showed similar concentrations with their normal-outcome counterparts. Compared to infected CB, infected MB showed higher concentrations of both pro-inflammatory cytokines (IL-2, IL-6, IL-7, TNF-α, IFN-γ), anti-inflammatory cytokine (IL-10) and chemokines (CCL2, CCL3) (Fig. 4). However, the concentration of CCL2 was elevated in CB compared with MB.

### Abnormal inflammatory responses in SARS-CoV-2 infected mothers

The laboratory measurements of blood routine at admission are listed in Tables 4 and 5. Compared with the COVID-19 negative pregnant women, the count of white blood cells (WBC) and neutrophils (NE) significantly higher in the COVID-19 positive pregnant women, while lymphocyte (LY) were lower in the COVID-19 positive pregnant women, which represented an infection status. In addition, COVID-19 positive women presented with higher rates of anemia and thrombocytopenia (Table 5). No significant intergroup differences were observed regarding aspartate aminotransferase (AST) and alanine aminotransferase (ALT) levels.

**Table 5:** Laboratory tests of COVID-19 positive and negative pregnant women before labor

Characteristic	COVID-19 positive *n* = 304	COVID-19 negative *n* = 910	OR(95%CI), *P*	OR*(95%CI), *P*
WBC > 9.5 × 10^9^/L, n (%)	130(42.8%)	282(31.0%)	1.664(1.274–2.173), 0.001	1.619(1.235–2.122), 0.001
NE%>0.75 × 10^9^/L, n (%)	136(44.7%)	267(29.3%)	1.950(1.492–2.547), 0.001	1.899(1.447–2.492), 0.001
LY%<0.2 × 10^9^/L, n (%)	189(62.2%)	374(41.1%)	2.355(1.804–3.075), 0.001	2.316(1.767–3.036), 0.001
RBC < 3.8 × 10^12^/L, n (%)	48(15.8%)	54(5.9%)	2.972(1.966–4.493), 0.001	2.805(1.841–4.274), 0.001
HGB < 115 g/L, n (%)	53(17.4%)	45(4.9%)	4.059(2.663–6.186), 0.001	3.807(2.474–5.857), 0.001
PLT < 125 × 10^9^/L, n (%)	15(4.9%)	18(2.0%)	2.572(1.280–5.169), 0.008	2.321(1.136–4.744), 0.021
AST > 35U/L, n (%)	5(1.6%)	19(2.1%)	0.784(0.290–2.118), 0.632	0.859(0.314–2.394), 0.766
ALT > 40U/L, n (%)	6(2.0%)	15(1.6%)	1.201(0.462–3.124), 0.707	1.195(0.454–3.149), 0.718

* Age, COVID-19 vaccination status, preterm labor, prelabor rupture of membranes, fetal growth restriction, postpartum hemorrhage, meconium-stained amniotic fluid, low birth weight (< 2500 g) and apgar score < 8 at 1-minute were adjusted.

Additionally, we segregated the COVID-19 positive group into a normal- outcomes group and an adverse-outcomes group. Only the count of WBC increased in the adverse-outcomes group compared to the normal-outcomes group (Table 6).

**Table 6:** Laboratory tests of COVID-19 positive pregnant women with adverse outcome or normal outcome before labor

Characteristic	Adverse outcomes *n* = 127	Normal outcomes *n* = 177	OR(95%CI), *P*	OR*(95%CI), *P*
WBC > 9.5 × 10^9^/L, n (%)	62(48.8%)	68(38.4%)	1.529(0.964–2.425), 0.071	1.648(1.018–2.668), 0.042
NE%>0.75 × 10^9^/L, n (%)	60(47.2%)	76(42.9%)	1.190(0.753–1.882), 0.457	1.203(0.749–1.932), 0.445
LY%<0.2 × 10^9^/L, n (%), n (%)	79(62.2%)	110(62.1%)	1.002(0.627–1.604), 0.992	0.854(0.644–1.702), 0.854
RBC < 3.8 × 10^12^/L, n (%)	24(18.9%)	24(13.6%)	1.485(0.800-2.757), 0.210	1.660(0.873–3.157), 0.122
HGB < 115 g/L, n (%)	26(20.5%)	27(15.3%)	1.430(0.789–2.592), 0.238	1.646(0.886–3.058), 0.115
PLT < 125 × 10^9^/L, n (%)	8(6.3%)	7(4.0%)	1.633(0.576–4.624), 0.356	1.598(0.551–4.632), 0.388
AST > 35U/L, n (%)	1(0.8%)	4(2.3%)	0.343(0.038–3.108), 0.342	0.473(0.050–4.459), 0.513
ALT > 40U/L, n (%)	1(0.8%)	5(2.8%)	0.273(0.032–2.366), 0.239	0.322(0.036–2.881), 0.322

* Age, place of residence, mode of delivery, gravidity, parity, medical comorbidities, and COVID-19 vaccination status were adjusted.

## Discussion

The results of this cross-sectional study provide evidence that SARS-CoV-2 infection in pregnant women during the third trimester leaded to overall adverse pregnancy outcomes and a unique humoral and cellular response at delivery. In SARS-CoV-2 infected pregnant women, only the concentration of IgG and IgM showed difference between adverse-outcome group and normal-outcome group, cytokines and immune responses were similar in two groups.

Large multicenter cohort study showed that pregnant women with COVID-19 infection had an increased risk of preterm birth and low birth weight [13, 14]. While opposite study suggested that there was no direct causal relationship between COVID-19 infection and maternal and neonatal poor outcomes except placental disorders [15]. In our study, the majority of SARS-CoV-2 infected pregnant women are asymptomatic or have mild symptoms. Still, the pooled prevalence of adverse pregnancy outcomes in COVID-19 positive women is 41.8%, which is higher than that observed in the general obstetric population. Preterm labor and Meconium-stained amniotic fluid were associated with SARS-CoV-2 infection. For the fetus, the incidence of low birth weight (< 2500 g) and Apgar score < 8 at 1-minute were markedly increased in newborns from COVID-19 positive mothers. Twenty-eight days after birth, the rate of low birth weight in neonates born by COVID-19 positive pregnant women with adverse outcome showed significantly difference compared to those with normal outcome pregnant women, suggesting that SARS-CoV-2 infection in the mother had an impact on the fetus. Our data revealed the maternal and neonatal outcome following a single exposure to COVID-19 in the third trimester. Previous studies described that several hundred cases of reinfection were mild, only a few cases were more severe [16]. There are few cases of reinfection during pregnancy. Verena et al. demonstrated that reinfection didn’t cause any adverse pregnancy outcome [17].

Previous studies have demonstrated that SARS-CoV-2 specific maternal antibodies can be transferred through the placenta during pregnancy and detected in the serum of the mother or their neonates after natural infection [18, 19]. IgM can be used for the diagnosis of early infection. The neonatal immune response is not fully mature, relying on the transplacental transfer of maternal antibodies in utero [20]. In our study, the RBD IgM antibody levels in CB serum were undetected. This undetectable IgM antibody in CB could be explained by the fact that the IgM antibody cannot cross the placenta owing to its large molecular weight. Nevertheless, few cases of vertical transmission of IgM have been detected in CB whose mother had severe COVID-19, indicating that the fetus could be infected with SARS-CoV-2 [21]. Early production of SARS-CoV-2 specific IgG antibodies is associated with progression from mild to severe COVID-19. In line with previous studies [22, 23], the presence of IgG in CB is mainly due to the passive transplacental transfer of this immunoglobulin from the mother to the fetus, we also observed an increment in RBD IgG antibody levels in the serum of both infected MB and CB, and there was a significant correlation between MB IgG antibody concentration and CB IgG antibody concentration. The transfer ratio were associated with duration between onset of maternal infection and time of delivery [19]. CB IgG can be transferred through maternal to the fetus, this maternal antibody transfer is a natural mode of immune transmission that helps protect newborns from the effects of viral infection.

SARS-CoV-2 infection induces cellular and humoral immune response, a paramount cause of severe manifestations of COVID-19 [24]. Cytokine storm, occurs due to dysfunctional immune response, inflicts multi-organ damage that leads to multi-organ failure [25, 26]. Garicia-Flores et al. confirmed the association of SARS-CoV-2 infection during pregnancy with humoral and cellular responses in the MB as well as with a mild cytokine response in CB [9]. We also observed increased concentrations of pro-inflammatory cytokines and chemokines, including IL-2, IL-6, IL-7, TNF-α, IFN-γ, CCL2, and CCL3 (MIP-1α) in the circulation of pregnant women with SARS-CoV-2 infection compared with paired CB. Secretions of such cytokines and chemokines participate in the recruitment and activation of immune cells to the infection site [27, 28]. We detected significantly higher inflammatory responses in COVID-19 positive pregnant women evident by increased IL-6 and TNF-α, which have been reported to induce the likelihood of preterm birth and can impact the development of the fetal neurological and respiratory system [29, 30]. Furthermore, increased levels of IL-6 upregulate IL-17. Notably, overexpression of IL-17 triggers the IL-17 receptor in fetal neurons, resulting in cortical dysplasia and behavioral abnormalities in fetuses [31, 32], and further augmenting the possibility of developing mental illness in adulthood [33]. Hence, obstetricians should be aware of the issues with early labor caused by the upregulated pro-inflammatory cytokines in SARS-CoV-2-infected pregnant women and even execute post-partum assessments and long-term follow-up to surveil the respiratory and neurological development of neonates. Among 12 cytokines, IL-7, G-CSF, CCL2, CXCL8 (IL-8) and CXCL10 are the prominent cytokines in CB. The primary function of IL-8 is to recruit and activate neutrophils to the infection site [34]. The reasons behind the different concentrations of cytokines in CB are complicated. On the one hand, cytokines have different abilities to cross the placenta barrier [35]. On the other hand, the levels of cytokines derived from intraplacental production are different [36]. Collectively, these findings strongly suggest that SARS-CoV-2 infection induces a remarkably maternal cytokine response and minimal neonatal inflammation.

Symptoms of COVID-19 vary in severity and presentation, from asymptomatic or mild symptoms to multiple organ failure and death [37]. The body’s defensive mechanism heavily relies on blood leukocytes. We observed significant changes in the hematological parameters in COVID-19 positive pregnant women. Our study demonstrated a marked increase in WBC/ NE and decrease in LY/RBC/HGB/PLT. Of note, anemia and thrombocytopenia is related with COVID-19 infection [38]. The proliferation of nucleated cells in bone marrow may be inhibited by SARS-CoV-2, leading to the marked decline in RBC and PLT counts in peripheral blood [39].

The data delineate detailed information on clinical and sociodemographic variables. However, the results of this study are subject to at least 4 limitations. First, the sample size was relatively limited since hospitalized pregnant patients constitute only a small portion of the infected population, and only 2 severe patients were observed in our study, which may result in the differences between the case and control group not being significant particularly. Second, as the samples were enrolled from a single center at the First Hospital of Jilin University, there is a risk of selection bias. Third, this observational cross-sectional study just reflected the situation of the territory during a peak period of the epidemic in 2023. Finally, Department of obstetrics in The First Hospital of Jilin University is the high-risk pregnant women treatment Center of Jilin Province, so the rate of cesarean delivery is higher than the general population. Despite these limitations, our data provided valuable insights into the unique immune responses triggered by SARS-CoV-2, which may help better understand the vulnerability of pregnant women to SARS-CoV-2 infection.

## Conclusion

Our study contributes to the knowledge that SARS-CoV-2 infection in pregnancy has been associated with significant adverse maternal peripartum outcomes. It also provides insight into the way the maternal-neonatal immune system responds to SARS-CoV-2 infection during labor. The findings of this study suggest that we need to focus more on SARS-CoV-2-infected pregnant women, a longitudinal observation should be done to assess long-term outcomes of their newborns. Future research is required to collect more comprehensive data to further corroborate these results, comprehend the pathophysiologic mechanisms behind these correlations, and identify effective strategies to avert unfavorable outcomes in COVID-19-positive pregnant women. Studies have shown that COVID-19 has a huge impact on pregnant women and babies, while the World Health Organization predicts that the X virus could harm humans at some point in the future. We think our study will provide the forward-looking forecasting data and models for the X virus infection in the future.

### Electronic supplementary material

Below is the link to the electronic supplementary material.

Supplementary Material 1

## Data Availability

No datasets were generated or analysed during the current study.

## Abbreviations

MB: Maternal blood
CB: Cord blood
G-CSF: granulocyte colony-stimulating factor
TNF-α: Tumor necrosis factor-α
CCL2: C-C motif chemokine ligand 2
CXCL10: C-X-C motif chemokine ligand 10
RBD: Receptor-binding domain
WBC: White blood cells
NE: Neutrophils
LY: Lymphocyte
AST: Aspartate aminotransferase
ALT: Alanine aminotransferase
