# Supplementary material for: Peripartum outcomes and immune responses after SARS-CoV-2 infection in the third trimester of pregnancy
Source: BMC Pregnancy Childbirth. 2024 Jul 24;24:498. doi: 10.1186/s12884-024-06707-5 (PMC11267945; doi:10.1186/s12884-024-06707-5)
Supplement: Supplementary file 1 — Supplementary Material 1 [file 12884_2024_6707_MOESM1_ESM.docx]

Survey for the study of “Peripartum outcomes and immune responses after SARS-CoV-2 infection in the third trimester of pregnancy”

Observational indicators

|  |  | Patient information |
| --- | --- | --- |
| Clinical characteristics | Maternal age |  |
|  | Place of residence |  |
|  | Mode of delivery |  |
|  | Gravidity |  |
|  | Parity |  |
|  | Medical comorbidities (diabetes, hypertension, cardiovascular, kidney, or lung disease) |  |
|  | COVID-19 vaccination status |  |
| Peripartum outcomes | Preterm labor |  |
|  | Meconium-stained amniotic fluid |  |
|  | Prelabor rupture of membranes |  |
|  | Fetal growth restriction |  |
|  | Postpartum hemorrhage |  |
|  | Adherent placenta |  |
|  | Oligohydramnios |  |
| Neonatal outcomes | Low birth weight |  |
|  | Apgar score <8 at 1-minute |  |
|  | Apgar score <8 at 1-minute |  |
|  | Neonatal fever |  |
| Laboratory tests | WBC% |  |
|  | NE% |  |
|  | LY% |  |
|  | RBC |  |
|  | HGB |  |
|  | PLT |  |
|  | AST |  |
|  | ALT |  |
